# Protein Expression Profile of ACE2 in the Normal and COVID-19-Affected Human Brain

**DOI:** 10.1021/acs.jproteome.2c00184

**Published:** 2022-07-28

**Authors:** Cecilia Lindskog, Loren Méar, Johan Virhammar, David Fällmar, Eva Kumlien, Göran Hesselager, Olivera Casar-Borota, Elham Rostami

**Affiliations:** † Department of Immunology, Genetics and Pathology, Rudbeck Laboratory, Uppsala University, 751 85 Uppsala, Sweden; ‡ Department of Neuroscience, Neurology, Uppsala University, 751 85 Uppsala, Sweden; § Department of Surgical Sciences, Radiology, Uppsala University, 751 85 Uppsala, Sweden; ∥ Department of Neuroscience, Neurosurgery, Uppsala University, 751 85 Uppsala, Sweden; ⊥ Department of Clinical Pathology and Cytology, Uppsala University Hospital, 751 85 Uppsala, Sweden

**Keywords:** SARS-CoV-2, COVID-19, neurology, ACE2, immunohistochemistry, proteomics, transcriptomics, antibodies, brain

## Abstract

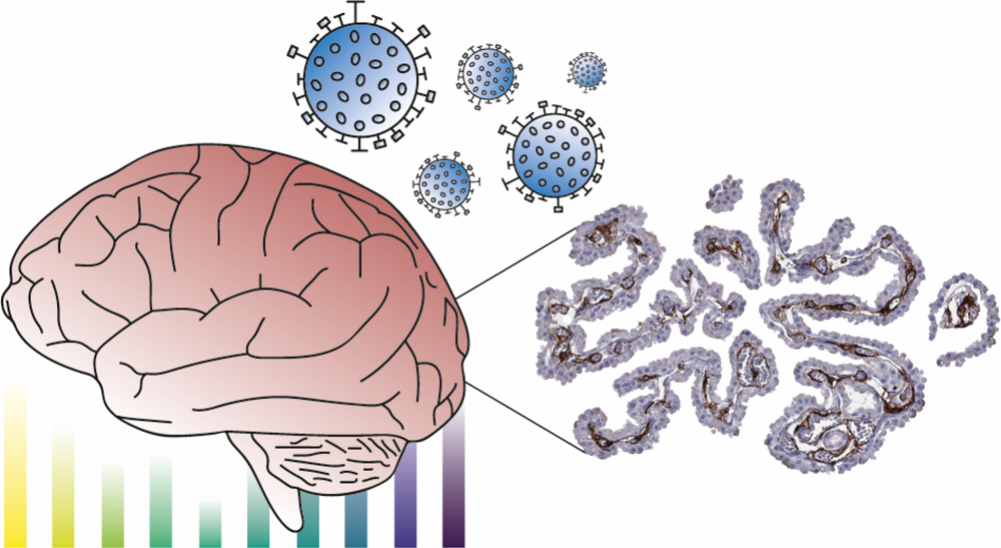

SARS-coronavirus 2 (SARS-CoV-2) that caused the coronavirus disease 2019 (COVID-19) pandemic has posed to be a global challenge. An increasing number of neurological symptoms have been linked to the COVID-19 disease, but the underlying mechanisms of such symptoms and which patients could be at risk are not yet established. The suggested key receptor for host cell entry is angiotensin I converting enzyme 2 (ACE2). Previous studies on limited tissue material have shown no or low protein expression of ACE2 in the normal brain. Here, we used stringently validated antibodies and immunohistochemistry to examine the protein expression of ACE2 in all major regions of the normal brain. The expression pattern was compared with the COVID-19-affected brain of patients with a varying degree of neurological symptoms. In the normal brain, the expression was restricted to the choroid plexus and ependymal cells with no expression in any other brain cell types. Interestingly, in the COVID-19-affected brain, an upregulation of ACE2 was observed in endothelial cells of certain patients, most prominently in the white matter and with the highest expression observed in the patient with the most severe neurological symptoms. The data shows differential expression of ACE2 in the diseased brain and highlights the need to further study the role of endothelial cells in COVID-19 disease in relation to neurological symptoms.

## Introduction

The COVID-19 pandemic caused by SARS-coronavirus 2 (SARS-CoV-2) has caused a major global challenge on healthcare as well as society. Evidence of neurological effects caused by the SARS-CoV-2 virus has been well established,^1^ which lead to, for example, encephalopathy,^2,3^ meningoencephalitis,^4^ demyelination,^5^ ischemic stroke,^6^ and brain hemorrhage.^7−9^ Not all patients with neurological symptoms end up in intensive care units, but due to the scale of the pandemic, these effects may still have a significant impact on both the individual and society as the symptoms may be long-lasting. The underlying mechanisms for different neurological effects are largely unknown, but they have been suggested to involve direct effects of the virus,^10^ secondary hyperinflammation syndrome,^11^ and immune-mediated disorders or result from a severe systemic disease.

One of the key proteins involved in SARS-CoV-2 host cell entry is the angiotensin I converting enzyme 2 (ACE2) receptor. While older studies suggested that the protein expression of ACE2 is relatively ubiquitous, more recent data utilizing stringent strategies for validation and, in particular, immunohistochemistry have shown that the expression of ACE2 is more restricted than previously thought.^12^ In a body-wide analysis of normal healthy tissues, ACE2 was not detected in the brain, but as the analysis of protein expression was based on tissue microarrays (TMAs) and a limited selection of samples, it is possible that a rare expression may have been missed.^12^ Recently, a collection of available datasets at the mRNA level^13^ proposed relatively high levels of ACE2 in the choroid plexus and paraventricular nuclei of the thalamus, but these findings have until date not been confirmed at the protein level. No previous study has performed immunohistochemistry of ACE2 across all major regions of the normal human brain. Furthermore, no study has looked at the distribution of ACE2 in COVID-19-affected brain tissue and compared the level of ACE2 expression with neurological symptoms.

In the present investigation, we performed a stringent immunohistochemical analysis to study the protein expression of ACE2 both in the normal human brain and in clinical samples from COVID-19 patients with various degrees of neurological symptoms. The protein expression patterns in the normal brain were also compared with data at the transcriptomic level.

## Materials and Methods

### Patients and Tissue Sample Preparation

Normal human brain and non-COVID-19-affected malignant glioma tissue samples for analysis of protein expression as well as the HPA tissue samples for analysis of mRNA expression were collected and handled in accordance with Swedish laws and regulations. The tissues were obtained from the Clinical Pathology department of Uppsala University Hospital, Sweden, and collected within the Uppsala Biobank organization. All samples were anonymized for personal identity by following the approval and advisory report from the Uppsala Ethical Review Board (ref nos. 2002-577, 2005-388, 2007-159, and 2011-473). The RNA extraction and RNA-seq procedure have been described previously.^14^

Postmortem COVID-19-affected brain tissue samples from three patients (Patient 1–3) and one surgical sample from a patient who underwent surgery for glioblastoma (Patient 4) were collected as part of a study approved by the National Ethical Review Authority (nos. 2020-01883 and 2020-02745) (Table S1). Informed consent was obtained from the patient undergoing tumor resection. The Declaration of Helsinki and its subsequent revisions were followed. All three patients were graded based on the Glasgow Coma Scale (GCS) ranging from 1 to 15 where GCS ≤ 3 means no response, GCS ≤ 8 means coma, and GCS = 15 corresponds to fully awake. The GCS was measured daily, and the values indicate the worst measurements. Both Patient 1 and 2 had severe brain injury with the worst GCS scores of 3 and 6, respectively, while Patient 3 had mild brain injury (worst GCS = 14). Based on histology (pathological examination of hematoxylin and eosin-stained sections), Patient 1–3 showed congestion (passive hyperemia) but none of the four patients had any signs of inflammatory cell infiltration, bleeding, infarction, or demyelination in any of the specimens. Patient 1 had signs of pronounced muscular weakness with the loss of tendon reflexes and was unconscious within one day after admission with EEG confirming status epilepticus. A CT scan of the brain revealed no new structural abnormalities, and a lumbar puncture showed no pleocytosis. The histology showed hypoxic cell damage in spread neurons found in the cerebral cortex, hippocampus, cerebellum, and pons. Patient 2 had a negative COVID-19 PCR test, but CT of the chest revealed bilateral pleural effusions and areas of ground-glass opacity with crazy paving patterns consistent with COVID-19 disease as confirmed by two independent radiologists. No additional COVID-19 diagnostic tests were performed since the patient was admitted in the beginning of the pandemic when clinical routines were not yet established. The patient deteriorated gradually in respiratory function. No focal neurological deficit was noted, and the CT of the brain showed only general cortical atrophy and unspecific white matter changes with no COVID-19-related findings; for example, there was no reduction in gray matter thickness. Lumbar puncture revealed no pleocytosis. The histology showed neuropathological changes associated with Alzheimer’s disease but no other histopathological findings. Patient 3 had poor respiratory function, and no imaging of the brain was performed. The histology showed moderate Alzheimer neuropathological changes as well as advanced synucleinopathy with the presence of Lewy bodies in neurons of the cerebral cortex and substantia nigra. As a control, we also stained a surgical sample of the brain from a COVID-19 patient that was admitted for resection of suspected glioma and tested positive upon admission (Patient 4). The patient did not experience any neurological symptoms (worst GCS = 15). CT and MRI showed an intraaxial tumor and unspecific subcortical white matter changes. Postoperative MRI showed expected findings without any signs of COVID-19-related manifestations. Histologically, the tumor was graded as glioblastoma WHO grade IV with a sparse amount of surrounding non-neoplastic brain white matter.

### Analysis of Transcriptomics Data

RNA expression data from HPA,^14^ GTEx,^15^ and FANTOM5^16^ as well as the normalized RNA expression dataset were retrieved from the HPA database (https://v20.proteinatlas.org). Consensus transcript expression levels were obtained through a normalization pipeline as described previously.^17^ For transcriptomic analysis in single cells, single nuclei Drop-seq (snDrop-seq) data (UMI count matrix) were downloaded for the cerebellar hemisphere and frontal cortex from the Gene Expression Omnibus (GEO) (https://www.ncbi.nlm.nih.gov/geo) database under series no. GSE97930.^18^ Data analysis was performed using Seurat v 4.0 (https://satijalab.org/seurat/)^19^ in R (CRAN). The analysis workflow, including the normalization, scaling, and clustering steps, has been previously described in Hikmet et al.^12^ Briefly, nuclei with <500 genes expressed were removed, and clustering was based on the 5000 mostly variable genes. Well-known marker genes were used to assign the cluster identity manually.

### Immunohistochemistry

For immunohistochemical analysis, formalin-fixed, paraffin-embedded (FFPE) tissue blocks of both normal and COVID-19-affected brain samples were sectioned, stained, and digitized essentially as previously described.^20^ Some of the normal brain samples as well as the malignant glioma samples were first assembled into tissue microarrays (TMAs) (Table S1). Paraffin blocks were cut in 4 μm sections using a waterfall microtome (Microm H355S, Thermo Fisher Scientific, Freemont, CA), placed on SuperFrost Plus slides (Thermo Fisher Scientific, Freemont, CA), dried overnight at room temperature (RT), and then baked at 50 °C for at least 12 h. Slides were immersed and boiled in citrate buffer, pH 6 (Lab Vision, Freemont, CA) for 4 min at 125 °C and then allowed to cool to 90 °C (the total program is approximately 40 min). Automated immunohistochemistry was performed by using a Lab Vision Autostainer 480S Module (Thermo Fisher Scientific, Freemont, CA) as described in detail previously.^20^ Primary antibodies toward human ACE2 were the monoclonal mouse IgG antibody AMAb91262, RRID: AB_2665871, (Atlas Antibodies AB, Bromma, Sweden) and monoclonal mouse IgG antibody MAB9331, (R&D Systems, Minneapolis, MN). All primary antibodies were diluted and optimized based on IWGAV criteria for antibody validation.^21^ For ACE2, normal kidney and small intestine tissues served as known positive controls, while tonsil, skeletal muscle, and skin constituted the negative controls as described previously.^12^ To determine potential unspecific binding of the secondary reagent, slides were also incubated with antibody diluent and secondary reagents only without the addition of primary antibodies. Protocol optimization was performed on a test TMA containing 20 different normal tissues. The glioma control samples were stained simultaneously with the other tissue samples. After addition of primary antibodies, the slides were further incubated with the secondary reagent anti-rabbit/mouse horseradish peroxidase-conjugated UltraVision (Thermo Fischer Scientific) for 30 min at RT and developed for 10 min using Diaminobenzidine (DAB) Quanto (Thermo Fisher Scientific) as a chromogen. All incubations were followed by rinsing in wash buffer (Thermo Fisher Scientific) two times for 5 min. Slides were counterstained in Mayers hematoxylin (Histolab, Gothenburg, Sweden) and cover-slipped using Pertex (Histolab) as a mounting medium. The stained slides were digitized with a ScanScope AT2 (Leica Aperio, Vista, CA) using a 20× objective. All tissue samples were manually annotated based on the staining intensity and fraction of positive cells using a three-graded scale: not detected (negative), low expression (weak staining or strong staining in <10% of the cells), or high expression (strong staining in ≥10% of the cells). For the normal tissues, a consensus score was set for each tissue type, taking all individual samples into consideration, while for the COVID-19 tissues, each individual was scored separately.

## Data Availability

All original high-resolution images of immunohistochemically stained tissue samples have been uploaded to the BioStudies repository (https://www.ebi.ac.uk/biostudies/).

## Results

### Transcriptomic Profiling of ACE2 in Human Brain Tissues

**Figure 1:**
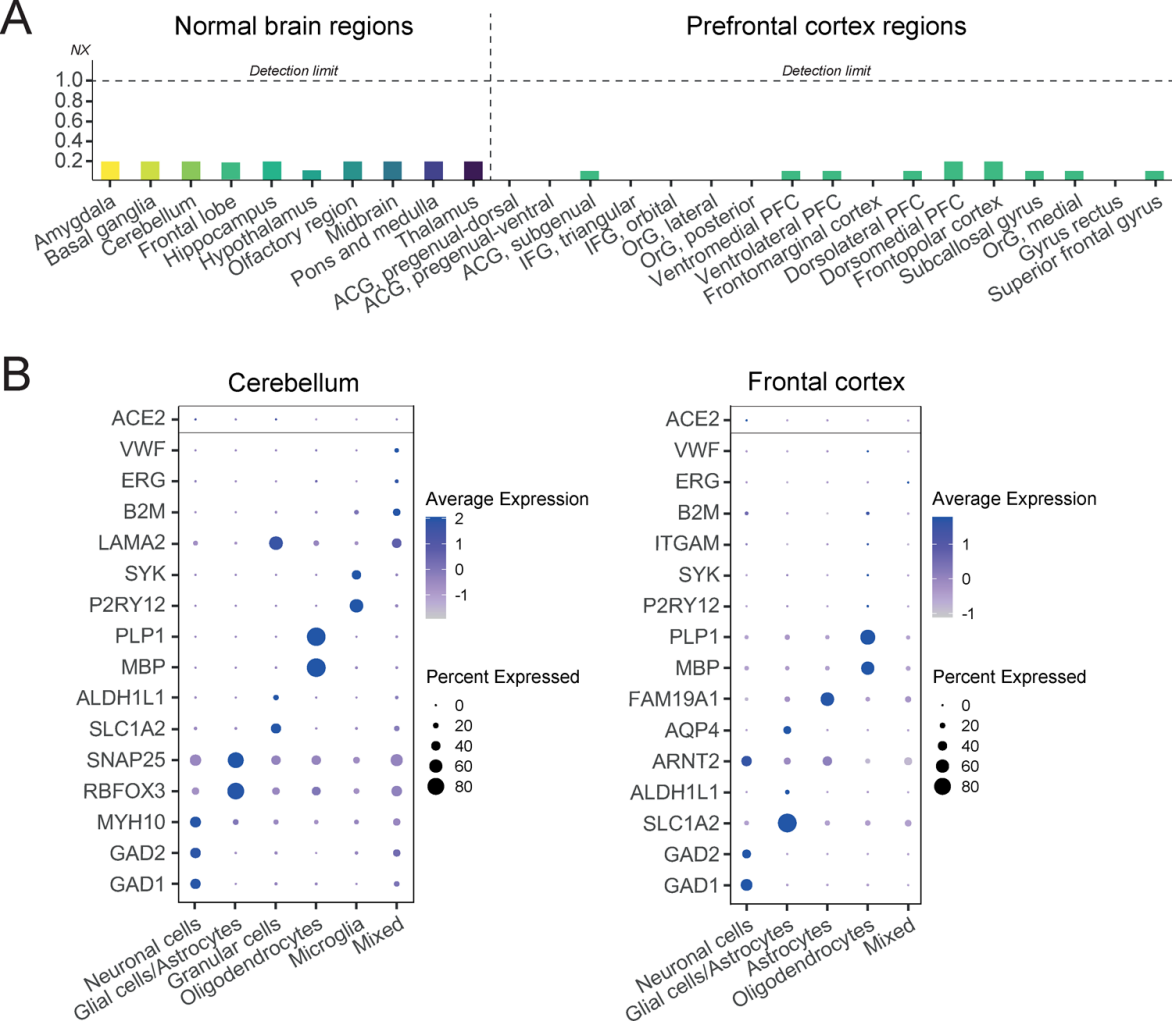
Transcriptomic profiling of ACE2 in the normal brain. (A) Overview of normalized expression levels (NX) of ACE2 based on bulk RNA expression in 10 normal brain regions as well as 17 different regions of the prefrontal cortex. For all regions, expression levels were below the detection limit. ACG = anterior cingulate gyrus, IFG = inferior frontal gyrux, OrG = orbitofrontal gyrux, and PFC = prefrontal cortex. (B) Analysis of ACE2 expression in the normal cerebellum and frontal cortex based on snRNA-seq. The expression of ACE2 (top row) was compared with known marker genes representing different cell types present in these tissues. No enrichment of ACE2 expression was observed in any of the analyzed cell types.

Several large-scale transcriptomics efforts provide a framework for quantifying the expression of protein-coding genes across different normal organs and tissues. Three such initiatives include the Human Protein Atlas (HPA) consortium,^14^ the genome-based tissue expression (GTEx) consortium,^15^ and the FANTOM5 consortium.^16^ In the HPA project, a combination of transcriptomics and antibody-based proteomics is used to characterize the entire human proteome, and we made the data publicly available in the open-access database www.proteinatlas.org. The HPA summarizes mRNA expression data from all three sources^22^ and merges the data into a consensus dataset, providing a comprehensive overview of transcriptomic levels in human tissues presented as normalized expression levels (NX).^17^Figure 1A shows an overview of ACE2 expression in the normal human brain based on transcriptomics with NX = 1 considered as the detection limit. The analysis covered 10 major brain regions and showed that the expression of ACE2 was below detection cutoff in all analyzed regions. Similar results are shown in a stand-alone detailed dataset generated by the HPA with 17 regions of prefrontal cortex, only displaying expression levels below the detection cutoff. We also analyzed the expression of ACE2 in single-nucleus RNA-seq datasets of the frontal cortex and cerebellum.^18^ No enrichment of ACE2 was observed in any of the analyzed cell type clusters (Figure 1B) as compared to the expression of well-known marker genes of cell types present in these tissues. The HPA project has also re-analyzed transcriptomics data from The Cancer Genome Atlas (TCGA) as part of the Pathology Atlas,^23^ which are presented as numbers of fragments per kilobase of exon per million reads (FPKM). Based on 153 glioma samples, the median expression of ACE2 was 0.1 FPKM with all samples showing an expression level below the detection limit, FPKM < 1.0 (data summarized on https://www.proteinatlas.org/ENSG00000130234-ACE2/pathology/glioma).

### Protein Profiling of ACE2 in Normal and COVID-19 Affected Brain Tissues

While transcriptomics has the advantage of quantitative measurements and low abundance detection, it is important to note that validation at the protein level is necessary to understand the role in health and disease as proteomics constitutes the functional representation of the genome. Spatial proteomics using immunohistochemistry has the advantage of determining the exact native localization in intact tissue samples. We used standardized immunohistochemistry for staining of ACE2 on 13 large sections and 22 tissue microarray (TMA) samples of the normal human brain corresponding to eight major brain regions. To investigate if the protein expression of ACE2 is altered in the COVID-19-affected brain compared to the normal brain, we used postmortem tissue samples from 9 to 12 different brain regions corresponding to three patients experiencing varying degrees of neurological symptoms (Patient 1, 2, and 3). As controls, we also included one COVID-19 positive patient without neurological symptoms that was admitted for glioblastoma (patient 4) as well as TMA samples from 11 individuals with malignant glioma that were not affected by COVID-19. The patient characteristics for the samples corresponding to COVID-19 patients are described in Table S1 and in the Materials and Methods, and all samples used are listed in Table S2.

**Figure 2:**
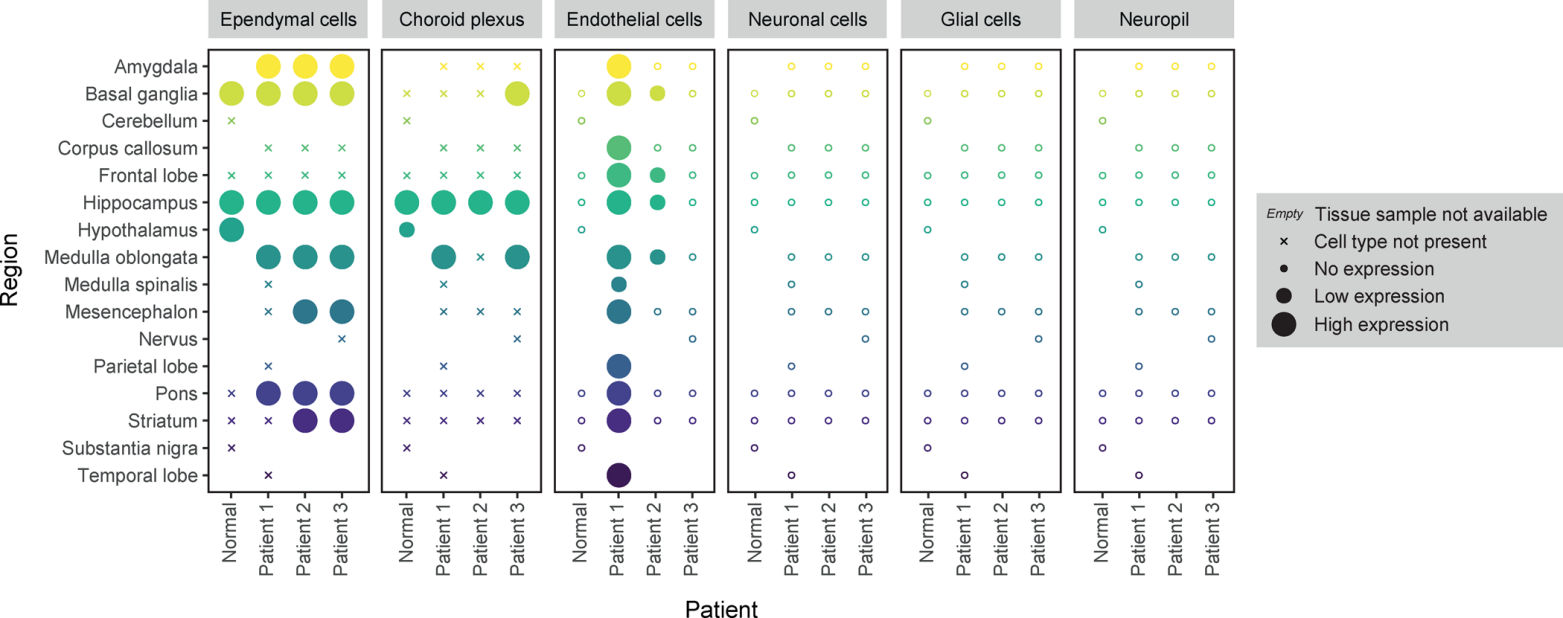
Protein profiling of ACE2 in normal and COVID-19-affected brain samples. Overview of protein expression levels in normal and COVID-19-affected brain samples based on immunohistochemical staining. In the normal brain, expression was observed in ependymal cells. A similar pattern of expression was found in COVID-19-affected brain samples, together with an upregulation of expression in endothelial cells in the subset of samples of Patient 1 and 2.

**Figure 3:**
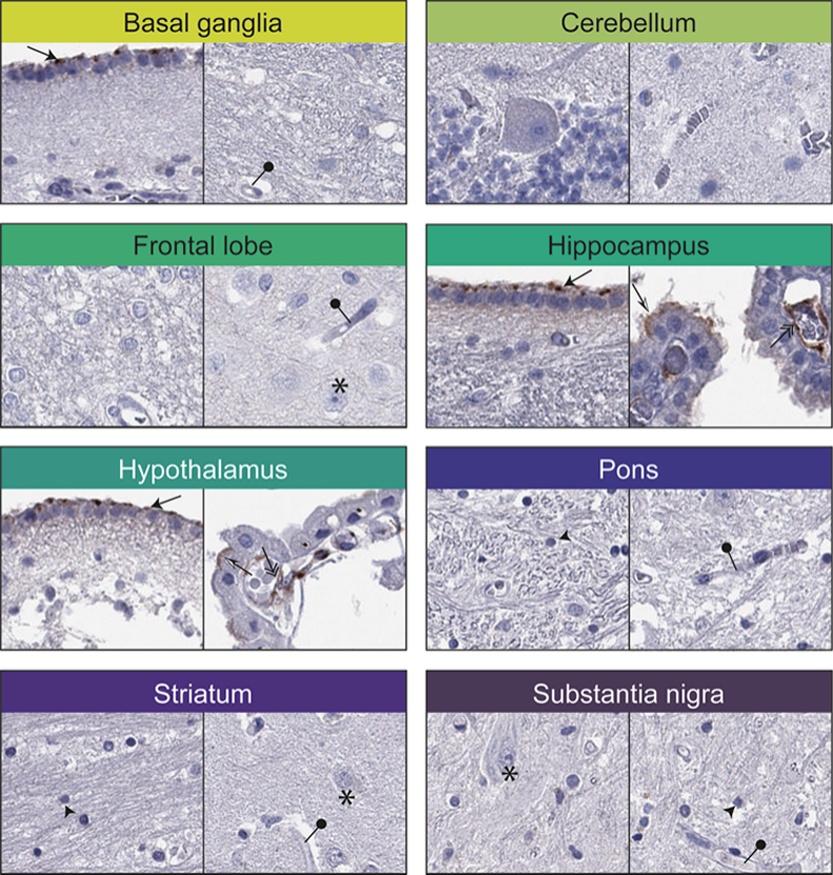
Immunohistochemical staining patterns of ACE2 in normal brain samples. Two representative images for each of the eight different regions of the normal brain. Distinct positivity was observed in ependymal cells (black arrows), choroid plexus cells (black and white arrows), and endothelial cells of the choroid plexus (double arrows). All other cell types, including neuronal cells (star), glial cells (black arrowhead), and endothelial cells in other brain regions (black needle), were negative.

Immunohistochemistry on normal and COVID-19-affected brain samples was performed using antibodies meeting stringent criteria for validation.^12^ An overview of the protein expression pattern across all brain regions is presented in Figure 2. In the normal brain, distinct expression was observed in a subset of ciliated cells in the ependyma and choroid plexus (Figure 3). In the choroid plexus, strong expression was also found in endothelial cells, but no positive endothelial cells were found in any of the other analyzed regions of the normal brain, neither in the white nor gray matter. Consistent with the single-nucleus RNA-seq dataset, no ACE2 protein was detected in neuronal cells, astrocytes, or oligodendrocytes in any of the brain regions.

**Figure 4:**
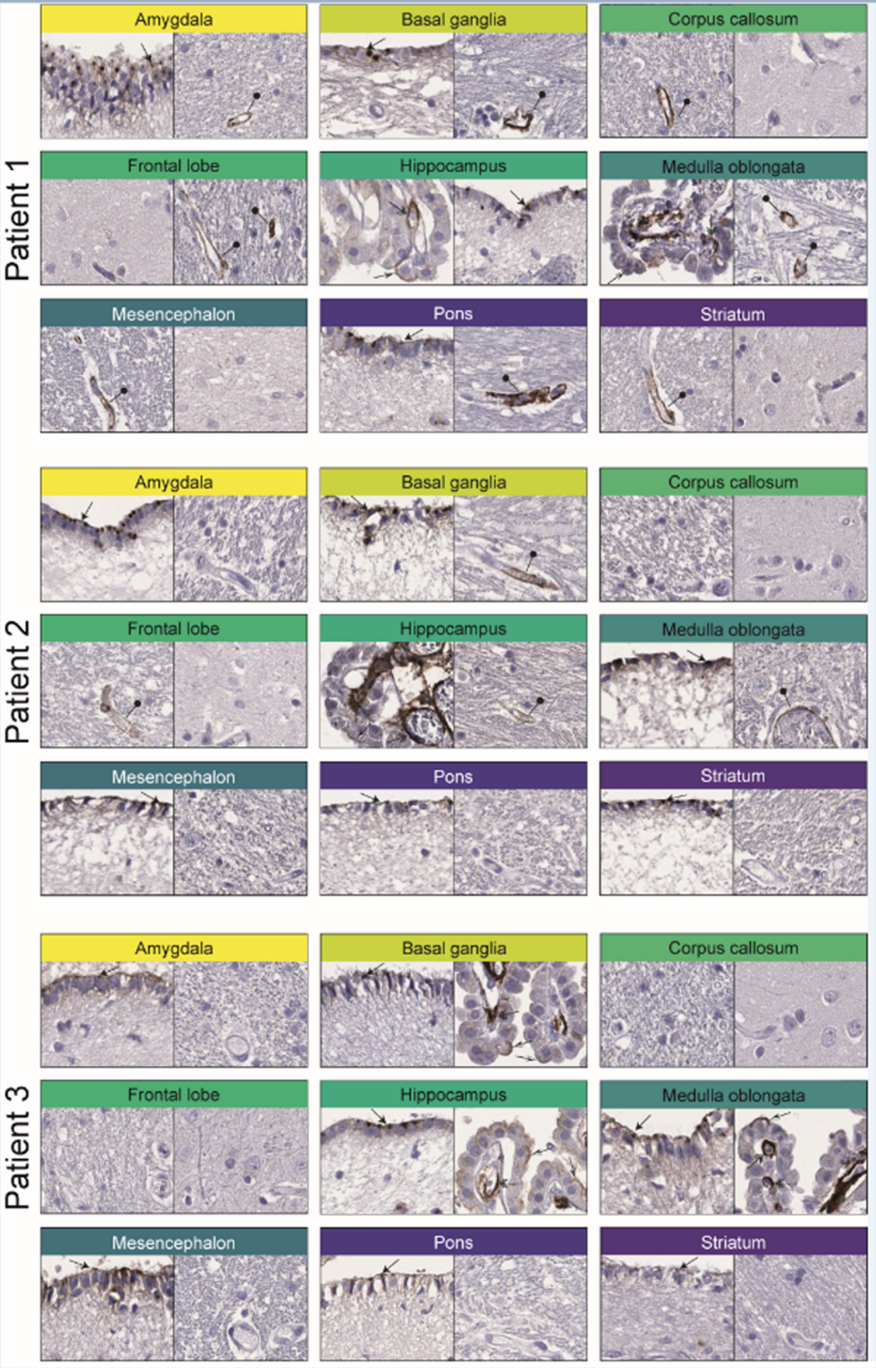
Immunohistochemical staining patterns of ACE2 in COVID-19-affected brain samples. Two representative images for each of the nine different regions of COVID-19-affected brain samples available for all three postmortem patients. Similar to the normal brain, distinct positivity was observed in ependymal cells (black arrows), choroid plexus cells (black and white arrows), and endothelial cells of the choroid plexus (double arrows). The expression of endothelial cells was upregulated in Patient 1 and 2 with the most abundant staining seen in Patient 1 generally in smaller vessels of white matter (black needle).

Similar to the normal brain, postmortem brain tissue samples from the three COVID-19 patients showed distinct protein expression in a subset of ciliated cells of the ependyma and choroid plexus as well as strong staining in endothelial cells of the choroid plexus (Figure 4). All three patients consistently also lacked protein ACE2 protein expression in neuronal cells, astrocytes, and oligodendrocytes. Interestingly, in contrast to the normal brain, both Patient 1 and 2, who showed the most severe neurological symptoms, displayed distinct ACE2 protein expression in a subset of endothelial cells (Figure 4). The endothelial cell staining was most prominent in the white matter and was, in general, both stronger and more commonly identified in Patient 1 compared to Patient 2. In Patient 3, who experienced milder neurological symptoms, no positive endothelial cells were identified in neither white nor gray matter in any of the analyzed samples. In order to confirm the upregulation of ACE2 expression in endothelial cells, a selection of normal samples and all COVID-19-affected brain samples were stained with a second independent antibody targeting non-overlapping sequences of ACE2, showing a similar pattern of expression (Figure S1).

In the control glioblastoma sample, endothelial cells were positive within the tumor cell compartment, but in contrast to the COVID-19-affected brain samples, no endothelial cell positivity was identified in the adjacent sparse non-neoplastic white matter (Figure S2a). For comparison, we also studied non-COVID-19-affected malignant glioma samples from 11 different individuals using both antibodies. One out of the 11 individuals showed positivity in a small subset of endothelial cells within the tumor cell compartment, while the remaining 10 individuals and all areas of adjacent non-neoplastic brain tissue were negative (Figure S2b). Thus, there was no general upregulation of ACE2 related to disease.

## Discussion

ACE2 is suggested to be a receptor for SARS-CoV-2 host cell entry, although alternative receptors have been proposed, ACE2 is still considered to be the key component crucial for infection. Previous studies have performed in-depth characterization of ACE2 expression at both the mRNA and protein levels from a body-wide perspective,^12^ finding little or no expression in the human brain. A low expression of ACE2 in the brain could however still be of major importance for the susceptibility of SARS-CoV-2 entry into the brain, a possible underlying mechanism of neurological symptoms. It should also be noted that a low expression level might be inconclusive as it is possible that the expression is higher in smaller structures localized to brain regions not previously studied or upregulated in COVID-19 disease. Most previous studies of ACE2 expression in the human brain have been performed on the mRNA level^13,24,25^ or were restricted to a limited sample collection,^12^ and no study compared the distribution of the ACE2 protein in the normal and COVID-19-affected brain based on immunohistochemistry. Here, we analyzed the cell type-specific localization of the ACE2 protein in the major regions of both the normal and COVID-19 human brain.

Antibody-based proteomics and immunohistochemistry is the main strategy for visualizing proteins in single cells in a spatial context and in relation to neighboring cells, a resolution not currently provided by other proteomics technologies, such as mass spectrometry. In immunohistochemistry studies, it is important to note that the antibodies must be properly validated before use in order to assure that the observed staining pattern corresponds to true protein expression and is not the result of unspecific binding. The International Working Group for Antibody Validation (IWGAV) has proposed different strategies for antibody validation to ensure reproducibility of antibody-based studies.^21^ It is emphasized that the validation must be performed in an application-specific manner.^26^ For immunohistochemistry, two main strategies are used: (i) orthogonal validation, which is defined as comparing the protein expression levels with an antibody-independent method analyzing the expression levels of the same target across tissues expressing the target protein at different levels, or (ii) independent antibody validation, which is defined as a similar expression pattern observed by an independent antibody targeting the same protein.^27^ In the present study, we used two independent antibodies that previously have been thoroughly validated by the HPA project. The immunohistochemical findings presented here in both the normal and COVID-19 affected brain were thus supported by independent antibodies targeting non-overlapping sequences of the ACE2 protein. Control TMAs containing 20 different normal tissue types were also stained in parallel as orthogonal validation, both serving as positive controls and assuring the absence of unspecific binding.

Here, ACE2 protein expression in normal brain was observed in ciliated cells of the ependyma and choroid plexus as well as in endothelial cells in the choroid plexus. The choroid plexus is an important structure for generation of cerebrospinal fluid (CSF)^28^ and serves as the blood–CSF barrier^29^ thereby constituting a possible entry point for the SARS-CoV-2 virus into the brain. The involvement of the choroid plexus in COVID-19 has previously been suggested due to high mRNA levels of ACE2 observed in microarray data^13^ and the ability of SARS-CoV-2 to infect the choroid plexus in human brain organoids.^30,31^ Protein-level expression or the exact cell-type distribution has however not previously been described.

The expression in ciliated cells of ependymal and the choroid plexus is consistent with the pattern observed in human airways where the ACE2 protein is restricted to a subset of ciliated cells in the upper respiratory tract,^12,32^ which is suggested as the main route of infection. We did not observe any ACE2 protein expression in neuronal cells, astrocytes, or oligodendrocytes in any of the analyzed brain samples. Similar results were previously obtained with immunohistochemistry based on TMAs,^12^ and the lack of expression in these cell types is supported by the general low or absent expression in the brain based on bulk RNA-seq as well as having no particular enrichment in any of the cell-type clusters in a single-nucleus RNA-seq dataset of the frontal cortex. Unfortunately, no single-nucleus data is available for ependymal cells or the choroid plexus, but the data included here confirms the lack of expression in the main cell types in the human brain. ACE2 protein expression in endothelial cells has previously been identified in several different normal tissues,^12^ but the data on endothelial cell expression in the human brain is somewhat contradictory. Some earlier studies have suggested staining in endothelial cells of the normal brain but without proper strategies for antibody validation, and it is thus not possible to determine if the observed staining in these studies correspond to true protein expression.^33^ Here, endothelial cell expression in the normal brain was restricted to the choroid plexus, which has not been investigated in any of the earlier immunohistochemistry studies. In other regions of the normal brain, no endothelial cell expression was observed, which is consistent with the results of seven different antibodies toward ACE2 in TMA samples from four main brain regions analyzed as part of the HPA project (https://www.proteinatlas.org/ENSG00000130234-ACE2/tissue).

In COVID-19-affected brain samples, we observed an upregulation of ACE2 in endothelial cells within the brain parenchyma with the highest expression observed in the patient with most severe neurological symptoms. ACE2 expression in endothelial cells of COVID-19-affected brain samples has previously been suggested in one patient of an autopsy study,^34^ but no study has compared the expression of ACE2 in relation to neurological symptoms. Upregulation of ACE2 in the diseased brain has been observed as a result of other conditions, for example, Alzheimer’s disease^35^ and hypertension,^36^ and in the present investigation, endothelial cells were distinctly stained within brain tumor cell compartments of a COVID-19 patient operated for brain tumor. No expression was however observed in adjacent brain areas showing normal histology. We also studied 11 additional malignant glioma samples from non-COVID-19-infected patients, out of which only two showed expression of ACE2 in a subset of endothelial cells in the tumor cell compartment. In this context, TMAs were used, and further studies using large sections are needed to fully investigate the potential heterogeneity of ACE2 expression in pathological samples to be able to conclude that there was no ACE2 expression in other areas not sampled. Nevertheless, there is no consistent general upregulation of ACE2 in the diseased brain and our study is the first one showing clear ACE2 expression in endothelial cells within histologically normal non-neoplastic brain tissue. We here show that ACE2 is consistently upregulated in endothelial cells throughout the brain in certain COVID-19 patients thereby highlighting a heterogeneity in expression between individuals that was not observed in any of the non-diseased brain samples.

Since only a few individuals were studied, it remains to be elucidated if the increased expression of the ACE2 protein in endothelial cells of certain COVID-19 patients is due to inter-individual variation caused by direct effects of the SARS-CoV-2 virus, which are triggered by mechanisms related to brain injury or other previous underlying diseases such as Alzheimer’s disease. One possible explanation is that host’s immune response may trigger an interferon-driven upregulation of ACE2, resulting in an increase in the number of cells susceptible for SARS-CoV-2 infection. This has previously been illustrated in the respiratory tract,^37^ but the exact role of SARS-CoV-2 in the human brain is yet to be understood. Nevertheless, the present investigation highlights specific cell types in the normal brain that express ACE2 and thereby constitute potential structures for SARS-CoV-2 entry in the human brain. Our study also shows that there is heterogeneity in expression of ACE2 in endothelial cells of diseased brain samples, which is displayed both in the COVID-19 samples and the samples from malignant glioma. This could indicate that mechanisms related to endothelial cells are involved in the development of neurological symptoms in COVID-19 disease. Since brain endothelial cells are the key constituents of the blood–brain barrier that protects the brain from pathogens and restricts access of circulatory factors, alterations in this barrier may be one of the explanatory factors leading to brain damage. Further large-scale studies on more patients with COVID-19 disease and a controlled sample collection with tissues from patients with various neurological diseases are urgently needed to gain a complete understanding of the underlying mechanisms of ACE2 expression in a diseased brain and its role in COVID-19-related neurological symptoms.

## Supporting Information Available

Immunohistochemical staining patterns of ACE2 in normal and COVID-19-affected brain samples based on independent antibodies, immunohistochemical staining patterns of ACE2 in glioma, overview of the patient characteristics for the four patients included in the study that were affected by COVID-19, and overview of the number of samples and sample type for all tissues included in the study (PDF)
