# Supplementary material for: Protein Expression Profile of ACE2 in the Normal and COVID-19-Affected Human Brain
Source: J Proteome Res. 2022 Jul 28;21(9):2137–45. doi: 10.1021/acs.jproteome.2c00184 (PMC9364976; doi:10.1021/acs.jproteome.2c00184)
Supplement: Supplementary file 1 — pr2c00184_si_005.pdf [file pr2c00184_si_005.pdf]

SUPPORTING INFORMATION

The protein expression profile of ACE2 in human normal and COVID-19 affected brain

Cecilia Lindskog[1*] Loren Méar[1], Johan Virhammar[2], David Fällmar[3], Eva Kumlien[2], Göran Hesselager[4], Olivera Casar-Borota[1,5], Elham Rostami[4]

[1] Department of Immunology, Genetics and Pathology, Rudbeck Laboratory, Uppsala University, 751 85 Uppsala, Sweden

[2] Department of Neuroscience, Neurology, Uppsala University, 751 85 Uppsala, Sweden

[3] Department of Surgical Sciences, Radiology, Uppsala University, 751 85 Uppsala, Sweden

[4] Department of Neuroscience, Neurosurgery, Uppsala University, 751 85 Uppsala, Sweden

5 Department of Clinical Pathology and Cytology, Uppsala University Hospital, 751 85 Uppsala, Sweden

Table of contents:

S-1: Figure S1. Immunohistochemical staining patterns of ACE2 in normal and COVID-19 affected brain samples based on independent antibodies.

S-2: Figure S2. Figure S2. Immunohistochemical staining patterns of ACE2 in glioma.

S-3: Table S2. Overview of the number of samples and sample type for all tissues included in the study.

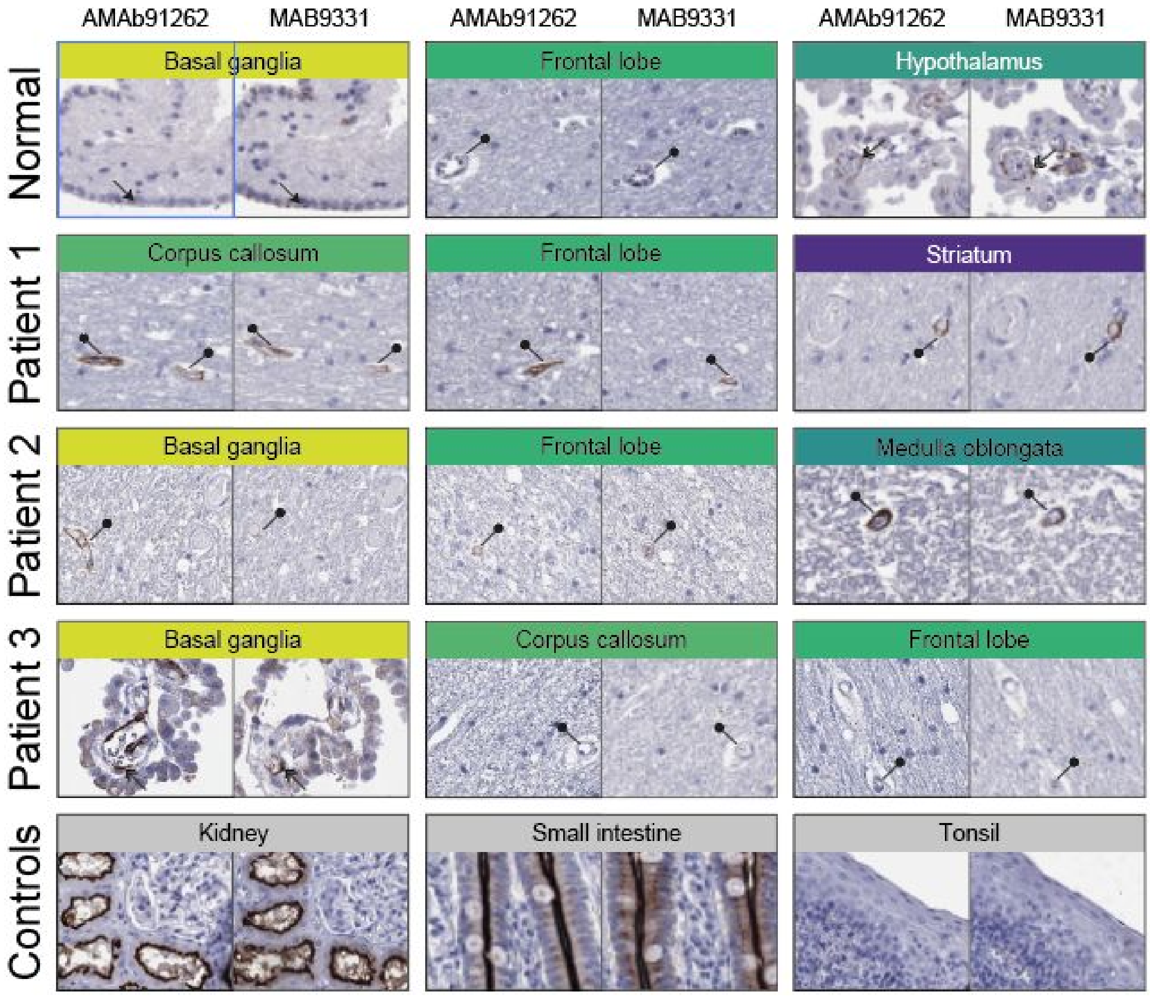

Figure S1. Immunohistochemical staining patterns of ACE2 in normal tissues and COVID-19 affected brain based on independent antibodies. Representative images corresponding to three different brain regions and three control tissue types based on consecutive sections stained with two independent antibodies; AMAb91262, Atlas Antibodies AB and MAB9331, R&D Systems. Based on both antibodies, ependymal cells (black arrows), and endothelial cells of choroid plexus (double arrows) stained in both normal and COVID-19 affected brain samples, while expression in other endothelial cells (black needle) was only observed in Patient 1 and 2. In the positive controls, both antibodies showed distinct staining in proximal tubules of kidney and microvilli of enterocytes in small intestine, while no staining

was observed neither in squamous epithelial cells nor immune cells in tonsil that served as negative control.

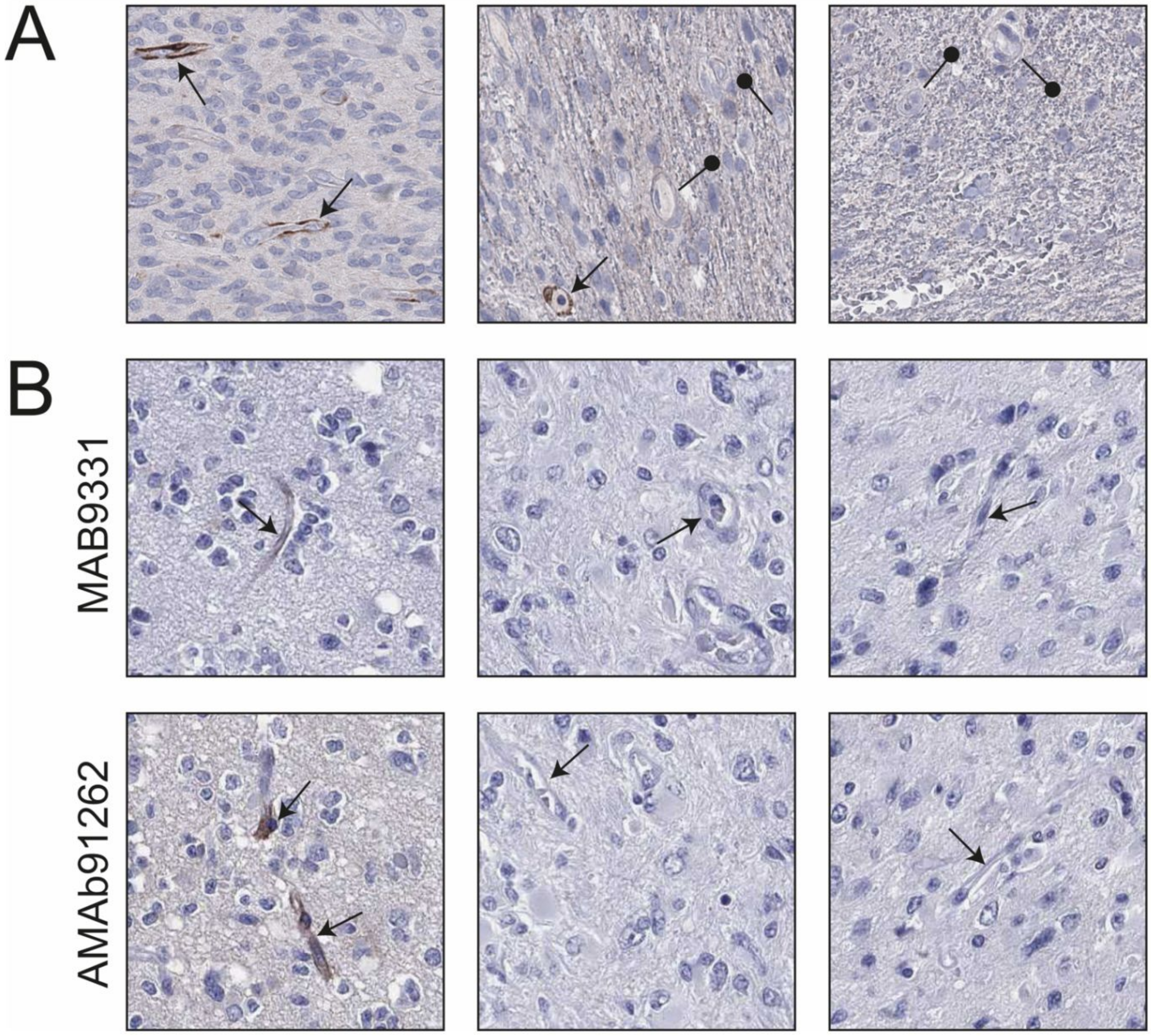

Figure S2. Immunohistochemical staining patterns of ACE2 in glioma. (A) Staining pattern in a COVID-19 affected glioblastoma sample. A subset of endothelial cells were distinctly positive within the tumor cell compartment (black arrows), but not in endothelial cells of adjacent non-neoplastic white matter (black needles). Left image represents tumor tissue, while middle image shows the transition zone between tumor cells (left part of the image) and non-neoplastic tissue (right part of the image). Right image represents normal histology. (B) Examples of staining patterns in three different non-COVID-19 affected glioma samples stained with two independent antibodies; AMAb91262, Atlas Antibodies AB and MAB9331, R&D Systems. Endothelial cells (black arrows) were positive in one individual only (left images).

Table S1. Overview of the patient characteristics for the four patients included in the study that were affected by COVID-19.

Table S1. Overview of the patient characteristics for the four patients included in the study that were affected by COVID-19.

Patient #	Age	Gender	Nasopharynx COVID-19 PCR test	Symptom to analysis (days)	Analysis to admission (days)	Adminssion to death (days)	Intensive Care Unit (ICU) (days)	Glasgow Coma Scale (GCS) worst	Confusion	Motor function	Respiratory assistance	WHO Score	Symptoms upon admission	Earlier diagnoses or risk factors
1	66	Female	Positive	12	3	10	10	3	1	1	Invasive ven	4	Dyspnea, high fever and somnolence	Pituitary adenoma, bipolar disorder, chronic obstructive lung disease and diabetes mellitus
2	84	Male	Negative	12	14	4	0	6	1	0	Oxygen mas	4	Cough, dyspnea and confusion	Heart failure and depression
3	82	Female	Positive	12	7	5	6	14	1	0	Invasive ven	4	Cough, fever and dyspnea	MGUS (IgA type) and chronic kidney failure
4	66	Female	Positive	20	0	N/A	0	15	0	0	None	1	Assymptomatic. Fever and anosmia one week prior to admission.	

Table S2. Overview of the number of samples and sample type for all tissues included in the study.

Region	COVID-19 affected brain large	Normal brain large section (n)	Normal brain TMA sample (n)	
Frontal lobe	3	0	0	
Temporal lobe	1	0	0	
Corpus callosum	3	0	0	
Parietal lobe	1	0	0	
Amygdala	3	0	0	
Hippocampus	3	3	5	
Striatum	3	0	2	
Basal ganglia	3	5	3	
Mesencephalon	3	0	0	
Pons	3	0	2	
Medulla oblongata	3	0	0	
Medulla spinalis	1	0	0	
Nervus	1	0	0	
Cerebral cortex	0	2	3	
Cerebellum	0	1	5	
Hypothalamus	0	2	0	
Substantia nigra	0	0	2	
**Brain tumor**	**Tumor compartment large section**	**non-neoplastic tissue large**	**Tumor compartment TMA sample**	**Non-neoplastmic tissue TMA**
Glioblastoma (COVID-19 affected)	1	1	0	0
Malignant glioma (non-COVID-19 affected) low grade	0	0	3	1
Malignant glioma (non-COVID-19 affected) high grade	0	0	8	1
